# Mental Health, Support System, and Perceived Usefulness of Support in University Students in Hong Kong Amidst COVID-19 Pandemic: A Mixed-Method Survey

**DOI:** 10.3390/ijerph191912931

**Published:** 2022-10-09

**Authors:** Agnes Y. K. Lai, George O. C. Cheung, Asa C. M. Choi, Man-Ping Wang, Polly S. L. Chan, Angie H. Y. Lam, Esther W. S. Lo, Chia-Chin Lin, Tai-Hing Lam

**Affiliations:** 1 School of Nursing, The University of Hong Kong, Hong Kong 999077, China; 2 School of Public Health, The University of Hong Kong, Hong Kong 999077, China

**Keywords:** mental health, university students, support system, resilience, family functioning, COVID-19, Hong Kong, university support

## Abstract

Background: This study explored the association of students’ mental health with their support system, identified the preferred ways and sources of support, investigated the perceived usefulness of available university support, and recommended actionable strategies to enhance students’ mental health. Method: An online questionnaire survey and semi-structured focus group interviews were conducted in 2021. Results: Among 1121 university students, 39.4% reported anxiety symptoms, which were less common in Chinese students and those pursuing medical and health programmes. Overall, 32.6% reported depression symptoms, which were more common in undergraduates. Both anxiety and depression symptoms were less common in students with higher resilience and support system and more common in students with family distress. Students with higher resilience had a better support system and less family distress. Perceived support from universities was lower than from peers and families. Peer support and phone contacts were the most preferred sources and ways of support. The most useful available university support was updated university guidelines, and the least useful was the emotional hotline service from universities The qualitative findings corroborated the quantitative results. Conclusion: We suggested that a holistic care approach and more proactive student-oriented university support would help students face adversity and enhance mental health.

## 1. Introduction

The unprecedented wave of the highly contagious Omicron variant of the novel coronavirus (COVID-19) was a disaster to the health system and society in Hong Kong. Since the first COVID-19 outbreak, the pandemic has impacted populations’ mental health [1]. University students have not only faced stress from adjustment to university environments, academic demands, and role transitions, but also additional challenges from frequent changes in school arrangement, government policy and uncertainties in career prospects during the pandemic [2,3,4]. High levels of mental distress, loneliness and poor quality of life were reported globally and locally [2,3,4,5,6,7].

A high-quality support system is essential for students’ mental health [8]. Researchers reported that students with better support had higher self-esteem and academic achievement, less emotional exhaustion, a better quality of life, and a better adjustment to adversity [5,9,10,11,12]. Masten et al. advocated the psychosocial adaptive capacities in the human system that comprised several levels, including individual, family, school, and community [13]. Similarly, Liu et al. proposed a dynamic, integrative, multi-level model of adversity coping, including individual, interpersonal and socio-ecological factors [14]. The individual factors refer to the trait-like characteristics of an individual. Resilience is one of the significant individual factors [15]. Students’ resilience not only promotes positive mental health well-being [16] but also contributes to academic achievement [17], positive coping styles [16] and life satisfaction [18]. Such resilience has been reported to indirectly mitigate the association between stress experience and acute stress disorder [19]. The interpersonal factors are the skills and resources from interpersonal experiences [14]. Family relationships are crucial for students’ mental health, particularly from early adolescence to midlife [20]. Good family functioning helps students relieve pressure from life challenges, safeguard their wellbeing [20,21], and it benefits their mental, physical and holistic development [22,23,24,25]. The socio-ecological factors range from institutional, community and societal factors. These factors operate within and across respective levels [14]. Modified from a multilevel model of adversity coping from Masten et al. and Liu et al., we explored the association between student mental health and a three-level support system, which includes individual, interpersonal and socio-ecological factors. The individual factors include student characteristics, study programme and resilience. The interpersonal factors include family functioning and support from peers and family members. The socio-ecological factors refer to socio-ecological contexts developed and facilitated over time [18]. In a higher education setting, university support, such as school connectedness, campus climate, faculty interaction, campus mental health services, barriers to accessing university support, material assistance and career counselling, were all associated with student mental health [26,27,28,29,30].

However, during the COVID-19 pandemic, many students reported a lack of quality support the schools, especially in the online learning environment [31]. Additionally, there is limited work adopting a multilevel approach systematically examining the factors influencing the mental health of university students during the COVID-19 pandemic and exploring students’ perception of the usefulness of support system. Only one qualitative study was found exploring how first-year students perceived students’ lives to affect their mental health [32]. Thus, the objectives of this study were to (i) explore the association of students’ mental health with a three-level support system, (ii) examine the association of students’ resilience with family functioning and support system, and (iii) reveal the preferred ways and sources of support, and the perception of the usefulness of university support from students’ perspectives. We hypothesised that it was less common for students with high resilience and good family functioning and support system to have anxiety and depression symptoms. The significance of this study was to report students’ perceptions and feedback on university support, highlight the insufficient perceived support from universities and provide important proactive actionable implications on university support. These might improve the effectiveness of university activities and support for enhancing students’ adversity coping and mental health.

## 2. Materials and Methods

### 2.1. Study Design and Participants

We used different types of information and communication technology strategies, from promotion and recruitment (by Facebook advertisement and instant messaging) to collecting quantitative data (by online survey platform “Qualtrics”) and qualitative data (by video conferencing “Zoom” focus group interview). Ethics approval was granted by the Institutional Review Board of The University of Hong Kong / Hospital Authority Hong Kong West Cluster (reference number: UW20-298). The inclusion criteria targeted university students aged 18 years or older studying in Hong Kong. Written informed consent was obtained from all students.

### 2.2. Procedures

#### 2.2.1. Collection of Quantitative Data

An online questionnaire, including items from well-validated scales and outcome-based questions, was distributed by an anonymous link generated by a Qualtrics survey tool with an exponential non-discriminative snowball sampling strategy from 7 October to 31 December 2020. During the survey, the new confirmed COVID-19 cases rose slowly from 8 to 54 cases per day [33]. Considering time sensitivity, snowball sampling was a cost-effective and efficient method to reach our study population [34]. Incorporating snowball sampling into social media, the survey link was first disseminated through Facebook advertisements and instant messaging platforms (e.g., WhatsApp) to recruit university students, which was a recognised and viable method of recruiting study participants not easily accessible or known to the researchers [35]. These students were encouraged to forward the survey link to their friends. To ensure all participants were university students studying in Hong Kong, we used two self-reported questions to ask students (i) whether they were studying at university and (ii) whether they were studying at universities abroad or in Hong Kong. Before participating in the survey, informed consent was obtained. Responses from repeated IP addresses were removed from the study to prevent duplicate entries. Upon completing the questionnaire, students received automatically computed scores with brief explanations for the scores of each validated scale in the questionnaire. Contact numbers and links for seeking help, support, or further reliable information on COVID-19 (e.g., link to the World Health Organization website) were provided. Students who completed the survey and left their contact number to us would automatically join a lucky draw to win video streaming (Netflix) subscription coupons (HKD 300 (USD38) for ten and HKD 400 (USD51) for two students) at the end of the study. The data file was encrypted and stored in a password-protected computer in a locked and secure location.

#### 2.2.2. Collection of Qualitative Data

Five one-hour online focus group interviews were conducted with randomly selected students who completed the questionnaire and agreed to join the interviews from 21 July 2021 to 28 July 2021. During those days, there were 1 to 3 new confirmed COVID-19 cases per day [33]. Before starting each focus group interview, the study’s objectives, purposes and design were explained clearly by the lead researcher (AL), a university academic and registered nurse with doctoral degrees in nursing and public health and 25 years of clinical nursing, teaching and research experience. A research assistant (AC) with a master’s degree in sociology took notes during the interviews to record important points mentioned by the interviewees. Another research assistant (GC) with a master’s degree in psychology monitored students’ responses to ensure that all of them actively took part in the interviews. Questions were structured chronologically to aid recall and were phrased to provide scope for additional issues to emerge. The questions focused on students’ experiences with COVID-19, the influence of their peer, family, and university support on their mental health and their perceptions of the usefulness of existing support. Written consent was obtained from participating students. The qualitative interviews were all tape-recorded and transcribed verbatim.

### 2.3. Measurements

#### 2.3.1. Exposure Measures

Resilience—A 2-item Connor–Davidson Resilience Scale-2 (CD-RISC-2) was used to assess adaptability and the ability to bounce back after hardship. Scores ranged from 0 to 8, with higher scores indicating better adaptability and resilience [36]. Scores at and below 4 indicate “low resilience”, and scores equal to 5 or above indicate “high resilience”. The Cronbach’s Alpha coefficient for the current sample was 0.70.

Family functioning—The 3-item Brief Assessment of Family Functioning Scale (BAFFS) was used to assess whether the participant agrees that their family can express feelings, get along well, and confide in each other. Scores ranged from 4 to 12. Scores at and below 6 indicate satisfaction with family functioning, and above 6 indicate distress [37]. The Cronbach’s Alpha coefficient for the current sample was 0.77.

Support system—The perceived support was assessed by asking three questions: “How would you rate the level of support from family/peers/universities during COVID-19?” on a scale of 0 (no support provided) to 10 (a lot of support provided). Higher scores in the three questions indicate more support. Scores of 5 or below indicate “low support”, and scores equal to 6 or above indicate “high support”. The one-item question has long been used in population surveys to measure one’s perception. There was a trend in survey research toward using the single global health status question to integrate the different dimensions of health [38]. Using a single item would minimise the burden on the students [39], would have the obvious advantage of brevity, making fewer demands than multi-item measures on students and would have obvious benefits for both research and policy in terms of reduced burden and costs, and ease of interpretation [40].

#### 2.3.2. Outcome Measures

Mental health—A 4-item Patient Health Questionnaire-4 (PHQ-4) was used to measure anxiety and depression symptoms. A shorter version of the screening scale was used to reduce the chance of missing data and improve efficiency [41]. The PHQ-4 includes the two-item Patient Health Questionnaire (PHQ-2) and the two-item General Anxiety Disorder screener (GAD-2). PHQ-2 has two DSM-IV diagnostic core criteria for major depression disorder, and GAD-2 has two criteria for generalised anxiety disorder that can also screen for panic and social anxiety disorders. Each item scores on a Likert scale of 0 (not at all) to 3 (nearly every day), with the total subscale scores ranging from 0 to 6. GAD-2 and PHQ-2 Scores of ≥ 3 are recommended to screen positive for anxiety and depression, respectively [41]. The Cronbach’s Alpha coefficient of the anxiety and depression subscales in the current sample were 0.84 and 0.77, respectively.

#### 2.3.3. Covariates

Sociodemographic and academic programme characteristics—Sociodemographic information included sex, age, ethnicity, country or residence. Academic programme characteristics included the mode of the programme, education programme level, programme year, the field of study, and whether the programme had a practicum component. Since we did not aim to compare the performance between universities, we did not ask which university the students were studying.

#### 2.3.4. Other Measures

Perceived usefulness of university available support—Students’ perceptions of support from universities that may be useful to themselves were assessed by 15-item in three aspects (informational (4 items), emotional (6 items), and instrumental support (5 items)). Response options included “Yes, it may be useful to me” and “No, it may not be useful to me”.

Preferred sources of support and communication—The preferred sources were assessed by the question, “Please select your preferred sources of support that may seek assistance when you experience emotional distress or problems”. Five response options: “peers”, “family”, “professional service”, “school and teachers”, and “spiritual group” were provided. One or more options could be selected. The preferred methods of communication were asked by the question, “Please select your preferred methods of communication to seek and receive advice from others.” Three response options, “face-to-face contact”, “social media platforms”, and “phone contact”, were provided, and one or more options could be selected.

### 2.4. Data Analysis

#### 2.4.1. Statistical Analysis

All quantitative analyses were performed with SPSS 26. All tests were two-sided, with *p* < 0.05 indicating statistical significance. Mental health problems refer to students having anxiety symptoms (GAD-2 scores of ≥ 3) and depression symptoms (PHQ2 scores of ≥ 3). Binary variables of anxiety and depression symptoms were the dependent variables in the following analyses.

First, we examined the differences in demographics and academic programme characteristics between students with and without anxiety and depression symptoms by logistic regression. Literature was shown that one’s perception and cognition might be affected by sex [42,43], age [44,45,46], and education level [42,46,47]. These might be the potential confounding factors. In our sample, our students ‘anxiety and depression symptoms had significant differences by ethnicity (Chinese versus non-Chinese), education programme level (undergraduate versus postgraduate), and field of study (medical or health-related programmes vs. no-medical or health programmes). Thus, sex, age, ethnicity, education programme level and field of study were adjusted in the following analyses. Second, we also used logistic regression to examine the differences in the level of resilience (“low” versus “high”), family functioning (“satisfaction” versus “distress”), and support from family (“low” versus “high”), peers (“low” versus “high”) and universities (“low” versus “high”) between those without and with anxiety or depression symptoms. Pearson product-moment correlation was conducted to examine the association among continuous variables of resilience, family functioning and support system. Third, we showed the proportion of students on their preferred sources of support, ways of communication, and perceived usefulness of available support from universities.

#### 2.4.2. Qualitative Analysis

The transcripts were coded and examined using Nvivo 11 by thematic analysis, adhering to the suggestions of Morse and Field [48]. Two researchers (G.O.C.C. and A.C.M.C.) independently reviewed the interview materials (including transcripts and recordings), summarised, and extracted meaningful statements. Field notes were reviewed during the analysis. Conflicting opinions on the contents of themes were discussed and resolved by a research group, including the lead researcher (A.Y.K.L.) and the other two researchers (G.O.C.C. and A.C.M.C.).

Different strategies were used to enhance the trustworthiness of the findings, including credibility (the truthfulness of data), dependability (the stability of data), confirmability (the congruence of data) and transferability (the applicability of data). To enhance study credibility, member checking was conducted by asking students (one student from each focus group interview) to review the transcripts from interviews they participated in and give feedback on emerging interpretations to ensure a good representation of their realities. Two researchers analysed each interview. Peer debriefing was then held to review the consistency of identified information with other co-investigators. To enhance study dependability, the description of the coding and the descriptions of themes were checked and reconfirmed by a research staff who was not involved in data collection. To promote study confirmability, an audit trail was conducted by making field notes when conducting interviews to allow tracing of the course of work. Most importantly, we reported the study design details, researchers, sampling strategies, data collection and analysis procedures to promote study transferability.

Mixed-method triangulation was adopted to interrelate and interpret the quantitative and qualitative data. The triangulation strategy assessed authenticity and trustworthiness through converging information from different sources [49].

## 3. Results

### 3.1. Participants

**Figure 1:**
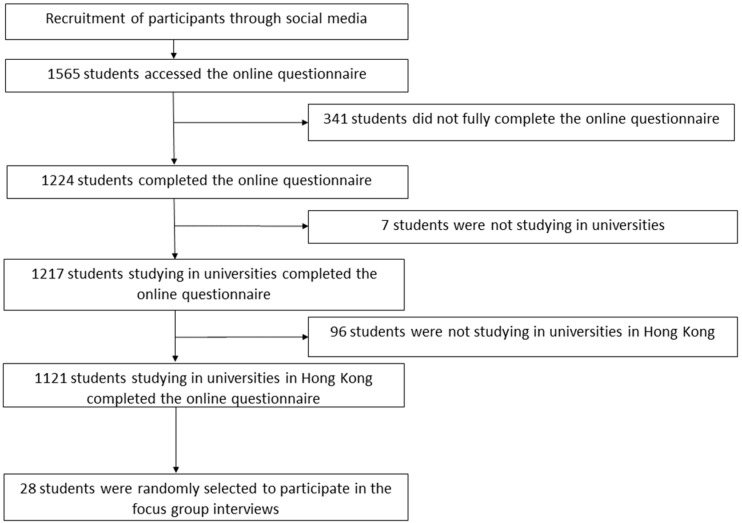
Recruitment flow chart.

A total of 1565 students accessed the online questionnaire. In total, 341 students were excluded, as they did not fully complete the questionnaires. Additionally, 7 students who were not studying in universities and 96 students who were not studying in universities in Hong Kong were excluded. A total of 1121 valid questionnaires were analysed in the current study (Figure 1).

Table 1 shows that 27.7% were males, and 80.8% were below 25 years old. Most were Chinese (91.6%) and Hong Kong residents (86.6%). Overall, 87% were full-time students, and 76.7% pursued a bachelor’s degree. Additionally, 30.8% and 23.7% were in their first and final year, respectively. In total, 43.3% were in medical or health-related programmes, and 49.5% of such programmes had practicum components. Twenty-eight students (57.1% females, 93% aged 18–34 years) were randomly drawn from those who participated in the survey and joined the five focus group interviews. All were Chinese, Hong Kong residents and pursuing undergraduate programmes. All conversations were audio-taped and transcribed verbatim.

### 3.2. Mental Health, Demographics and Academic Programme Characteristics

Table 1 shows that the mean scores of anxiety and depression symptoms were 2.6 ± 1.5 and 2.1 ± 1.5; 39.4% and 32.6% of students had symptoms of anxiety and depression, respectively. Table 2 shows that anxiety symptoms were less common in Chinese students than other ethnicities (38.1% versus 54.3%, *p* = 0.002, adjusted odds ratio (aOR) = 0.52, 95% confidence interval (C.I.) = 0.34–0.79), and anxiety symptoms were less common in students pursuing medical or health-related programmes than those pursuing other programmes (33.4% versus 44.0%, *p* < 0.001, aOR = 0.64, 95% C.I. = 0.50–0.81). Depression symptoms were more common in undergraduate students than postgraduate students (34.3% versus 26.8%, *p* = 0.02, aOR = 1.42, 95% C.I. = 1.05–1.94). No significant differences in sex, age, country and region of residence, programme year and programme with a practicum component were found between students with and without symptoms of anxiety and depression.

### 3.3. Resiliences

Table 1 shows that the mean resilience score was 6.1 ± 1.8 (out of 8), and 48.1% of the students had high resilience. Table 3 shows that it was less common for students with high resilience to exhibit anxiety symptoms (28.2% versus 49.8%, *p* < 0.001, aOR = 0.39, 95% C.I. = 0.30–0.50) and depression symptoms (19.9% versus 44.3%, *p* < 0.001, aOR = 0.31, 95% C.I. = 0.23–0.40) than students with low resilience. Table 4 shows the significant associations of resilience with family functioning and support system with or without adjusting for potential confounders (all *p* values < 0.001).

### 3.4. Family Functioning

Table 1 shows that the mean family functioning score was 6.1 ± 1.8, and 34.6% of the students had family distress. Table 3 shows that it was more common for students with family distress to report anxiety symptoms (49.0% versus 34.4%, *p* < 0.001, aOR = 1.90, 95% C.I. = 1.47–2.54) and depression symptoms (43.6% versus 26.7%, *p* < 0.001, aOR = 2.15, 95% C.I. = 1.65–2.79) than students with better family functioning.

### 3.5. Support from Family and Peers

Table 1 shows that the mean scores of perceived support from family and peers were 6.7 ± 2.6 and 6.3 ± 2.4, respectively. Additionally, 70.7% and 66.6% of the students reported high perceived support from family (high family support) and peers (high peer support), respectively. Table 3 shows that it was less common for students with high family support to exhibit anxiety symptoms (37.1% versus 45.0%, *p* < 0.01, aOR = 0.67, 95% C.I. = 0.52–0.88) and depression symptoms (29.9% versus 38.9%, *p* < 0.01, aOR = 0.66, 95% C.I. = 0.51–0.87) than students with low family support. Similarly, it was less common for students with high peer support to exhibit anxiety symptoms (35.2% versus 47.9%, *p* < 0.001, aOR = 0.58, 95% C.I. = 0.45–0.75) and depression symptoms (29.2% versus 39.3%, *p* < 0.001, aOR = 0.63, 95% C.I. = 0.49–0.82) than students with low peer support.

In the focus group interview, students stated that their family conflicts increased and their emotion was adversely affected by increased low-quality contact time with family members. Students also reported that they enjoyed online time with peers, which was better than studying alone.

*“For instance, as my family members and I stayed at home longer, more family fictions arose, which made my mood fluctuate and affected my study”.*

(Participant 11, female, 23 years old, Bachelor programme, major in Medicine)

*“If I were sitting around and doing nothing at home, they (my parents) would wonder why I just sat there and didn’t study and considered me wasting time. They didn’t understand the mode of study in university”.*

(Participant 12, Female, Bachelor programme, Major in Chinese)

*“I turned on zoom with my peers when doing my homework and chit chat. I enjoyed the time with my classmates, which was better than staying home alone”.*

(Participant 11, female, 23 years old, Bachelor programme, major in Medicine)

### 3.6. Support from Universities

Table 1 shows that the mean score of perceived university support (university support) was 4.4 ± 2.5, and only 32.3% of the students reported receiving high university support. The university support score was significantly lower than the peer support (mean 4.4 versus 6.3, *p* < 0.001) and family support scores (mean 4.4 versus 6.7, *p* < 0.001).

Table 3 shows that it was less common for students receiving high university support to exhibit anxiety symptoms (35.4% versus 41.4%, *p* < 0.01, aOR = 0.77, 95% C.I. = 0.59–1.0) and depression symptoms (25.7% versus 35.8%, *p* < 0.01, aOR = 0.62, 95% C.I. = 0.47–0.83) than students receiving low university support.

In focus group interviews, similar findings of insufficient support from the universities were reported.

*“I think the opening hours of the facilities can be longer. For instance, learning centres and libraries can be opened 24 hours a day”.*

(Participant 16, male, 20 years old, Bachelor programme, major in History)

*“A friend of mine can’t access WIFI at home, and I saw him taking the exam on a bench at the public area on the university campus. School should provide more facilities to them”.*

(Participant 1, female, 22 years old, Bachelor programme, major in Engineering)

*“I think our university can provide more clear instruction on the course arrangement and guidelines on examination. The unclear instruction made me feel stressed”.*

(Participant 1, female, 22 years old, Bachelor programme, major in Engineering)

*“It was difficult to follow when using a longer video for learning and revising”.*

(Participant 16, male, 20 years old, Bachelor programme, major in History)

*“I felt a bit helpless and loss, especially I was a new student. I did have any face-to-face or phone communication with teachers. Everything was connected by emails and was robotics”.*

(Participant 16, male, 20 years old, Bachelor programme, major in History)

### 3.7. Preferred Sources of Support and Ways of Communication

**Figure 2:**
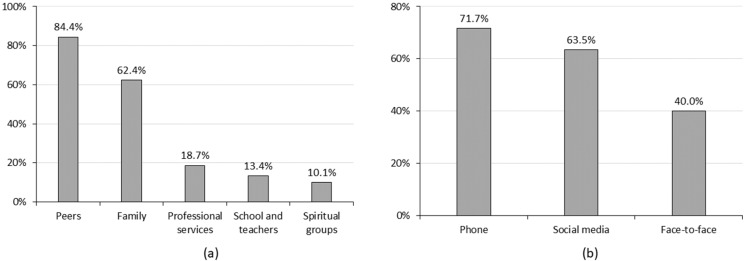
The preferred sources of support and ways of communication. (**a**) Preferred sources of support; (**b**) preferred ways of communication.

Figure 2 shows that the top three most preferred sources of support for students during the COVID-19 pandemic were supported from peers (84.4%), family (62.4%), and professional services (18.7%). Only 13.4% preferred to seek support from their universities. Additionally, 71.7% of the students preferred phone contact, followed by contact through social media platforms (63.5%) and face-to-face contact (40.0%).

### 3.8. Perceived Usefulness of University Support

**Figure 3:**
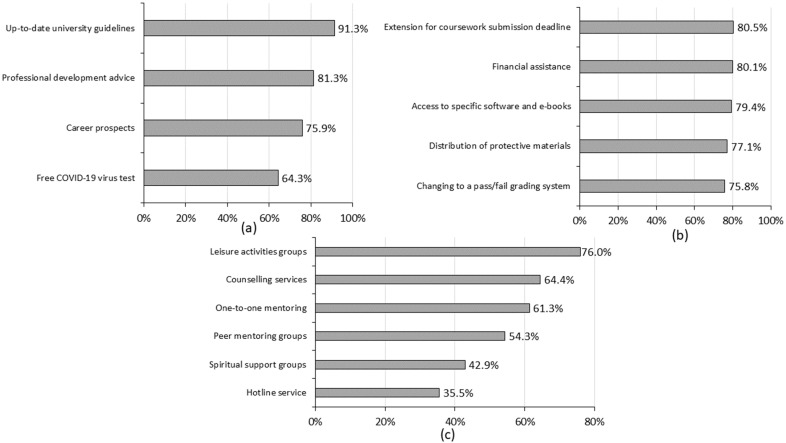
The perceived usefulness of support from universities. (**a**) Informational support; (**b**) instrumental support; (**c**) emotional support.

Figure 3 shows students’ perception of university support that might be useful to them (perceived usefulness). Regarding informational support, the top three pieces of information perceived to be useful were information on up-to-date guidelines and arrangements from universities (91.3%), followed by personal development advice (81.3%) and information on career prospects (75.9%).

In the focus group interview, many students reported feeling stressed because of the confusing lecture schedules and unclear examination timetables and format. Thus, up-to-date guidelines and arrangements from universities could help relieve their anxiety.

*“… (I) was quite stressful. (I) studied in year two at that time. All classes had been cancelled, and we didn’t hear any arrangements from the faculty. I remembered that the faculty informed us that the examination would be held in July at the beginning. Still, suddenly, they announced changing the examination to June and May”.*

(Participant 19, Female, 21 years old, Bachelor programme, major in Medicine)

*“How to attend the (online) lectures, modes of lessons, the schedule of classes. The arrangement was confusing… updated guidelines and information can help me better prepare and relieve my stress and anxiety”.*

(Participant 22, Male, 29 years old, Master’s programme, major in Environmental science)

Regarding instrumental support, an extension of coursework (80.5%) was perceived as the most useful support, followed by financial assistance (80.1%) and the accessibility of e-books and specific software for self-learning (79.4%).

In the focus group interviews, students reported similar findings that the main source of stress still came from their assignments. The improved accessibility of e-resources enriched their knowledge and boosted their learning efficiency.

*“There were many assignments to submit within the same week. It was stressful”.*

(Participant 3, Male, 18 years old, Bachelor programme, major in Engineering)

*“I had read more academic papers this year than what I have done in the past two years”.*

(Participant 19, Female, 21 years old, Bachelor programme, major in Medicine)

*“I can have more time to attend various virtual conferences and webinars and learnt more from different experts. I enjoyed this good opportunity”.*

(Participant 21, Female, Major in Environmental Science)

Regarding emotional support, the leisure activities groups (76.0%) were the most useful support, followed by counselling services (64.4%) and one-to-one peer mentoring (61.3%). The least useful support from universities was hotline services (35.5%).

In a focus group interview, students reported they preferred to talk with peers, did not like hotline service, and were disappointed with discontinuing all non-academic-related activities.

*“If I feel not happy, I preferred to talk with my friends and seldom made a call to hotline because I think it would not help much”.*

(Participant 19, Female, 21 years old, Bachelor programme, major in Medicine)

*“Joining extracurricular activities might help to stress from the study. However, most of the activities and field trips were discontinued because of the outbreak”.*

(Participant 21, Female, Major in Environmental Science)

*“I have no confidence in the hotline services. From my friends’ experience, the suggestion from hotline services was too general. Besides, it took time for university to response to her issues”.*

(Participant 1, female, 22 years old, Bachelor programme, major in Engineering)

## 4. Discussion

Our study systematically reported the association of students’ mental health with a three-level support system and suggested key actionable strategies for enhancing students’ mental well-being, especially amidst the COVID-19 pandemic.

Our study reported that anxiety symptoms were less common in Chinese students than non-Chinese students, which might be explained by the additional challenges encountered by non-Chinese students. With ethnic Chinese being the dominant group in Hong Kong, non-Chinese groups might need to assimilate into the local culture by adapting and integrating with the majority of the community [50]. Some non-Chinese international students might face more challenges living abroad, adjusting to the host country’s culture and norms, and being away from central social support system [5,51]. Our findings also showed that it was common for students pursuing non-medical and health-related programmes to have anxiety symptoms, which might be due to better knowledge of the pandemic in medical and health-related students than students studying other programmes. However, inconsistent findings were reported by a meta-analysis, showing a relatively higher prevalence of depression and anxiety among medical students during COVID-19 than in the general population [52]. Such differences might relate to continuing medical internship training in other countries. However, all clinical practicum of medical and health-related programmes in Hong Kong was discontinued during the survey. Additionally, depression symptoms were more common for undergraduate students than postgraduate students, which was consistent with the pattern shown in the Student Experience in the Research University Consortium survey [53]. The lower mental health of the undergraduate students before [54] and during [55] the pandemic might be explained by the less life experience in undergraduate students.

Resilience, family functioning and support system are essential to help students adapt to drastic changes in life and academic challenges. Symptoms of anxiety and depression were less common for students with better resilience, family functioning and support system; similar findings were reported in other studies [16,56]. Family functioning might alleviate students’ mental health and enhance positive communication [57,58]. Additionally, our samples showed positive associations of perceived peer and university support (such as school measures) with mental health, which was consistent with the findings in the literature. [7,26]. However, the significantly lower support from universities than support from peers and family has not been discussed in other studies.

The study’s strength was to include qualitative and quantitative data to enrich the understanding of the mental health of university students amidst the pandemic. Our work highlighted the insufficient perceived support from universities and provided important actionable implications for academic institutions to strengthen the support system of university students. A single solution cannot address the complexity of students’ mental health. Related factors from different levels of support system should be considered to better understand the complex interplay between students’ mental health and their support system.

Currently, most university support and policies in Hong Kong focus on infection control, such as temperature check in all indoor locations, vaccine pass, quarantine arrangements, and reporting campus-related cases. We recommend that universities should adopt a holistic approach to education by implementing initiatives to meet students’ learning and social and emotional needs and be more proactive to enhance student health. To accommodate the learning needs of students, first, for disadvantaged and vulnerable students, distributing free electronic devices, providing internet connection, and unlocking or creating emergency funds as financial support would be the most direct and effective support. Second, universities should help students catch up on missing learning and provide more proactive support (such as supplementary classes or consultation sessions) based on individual needs and preferences. Third, apart from mental health services, university policies also played a significant role in mitigating students’ distress by the swift and efficient dissemination of updated arrangements of classes, tutorials, practicums, and learning facilities. Fourth, various formative and summative assessment schemes should be flexibly adjusted to align with current pandemic conditions. Innovative e-learning tools and resources should be further promoted in the current unique circumstance to facilitate seamless transfer of knowledge to students and even enrich the student learning experience in universities. To meet students’ social and emotional needs, activities could be designed to strengthen students’ adaptive capacity for both emotion-focused and problem-focused coping. Proactive counselling services and integrating the concepts of specific positive psychology themes (e.g., savouring and gratitude) into the teaching and learning curriculum might cultivate positive coping toward life challenges. Providing extra-curricular activities such as mindfulness Zentangle drawing might ameliorate students’ anxiety and depression symptoms [59]. Lastly, providing online resources and designing training to support teachers in teaching could enhance the quality of university support. In future research, we could examine the effectiveness of our suggested intervention and integration in improving mental health.

The study had several limitations. First, the data collected through an online survey might be subject to self-selection bias as the most vulnerable could not be reached; the lucky draw incentives offered in this study might have selection bias, as some students did not interest in the video streaming coupons. Second, using non-random snowball sampling to recruit suitable students efficiently could result in sampling bias from students forwarding the survey to peers with similar traits and characteristics [34]. Thus, we used a wider range of e-platforms with different media to disseminate the survey to reach students with different demographic characteristics and universities. Third, the percentage of students who answered the online survey was higher in females than males. Females responding at much higher rates than males are common [60]. Females are more likely to engage in online activity characterised by communication and exchanging information. In contrast, males are more likely to engage in online activity characterised by information-seeking [61]. Fourth, because validated questionnaires were unavailable for measuring students’ support system related to the COVID-19 pandemic, we developed our outcome-based questions to assess the support system of family, peers and universities. Nevertheless, the quantitative findings were corroborated by the qualitative findings from focus group interviews. Fifth, although the different support sources included in our survey may be popular amongst students, the list was not exhaustive. Popular support might not be the most effective in protecting against adverse mental health problems. Sixth, the current study did not collect detailed information on whether students had been exposed to the support. Thus, it was unable to compare the difference in perceived usefulness of university support between those students who received university support and those who did not receive it. Seventh, although we found non-Chinese students have lower mental health than Chinese students, we did not include non-Chinese students in the focus group interviews to further explore the reasons and affecting factors. In future research studies, we should collect more in-depth information to further investigate the difference in the factors and mediators affecting mental health between Chinese and non-Chinese students. Lastly, the generalizability of our results could be limited due to the snowballing sampling method, and non-Chinese university students were not included in the focus group interview, which might cause sampling bias.

## 5. Conclusions

To conclude, good resilience and a strong support system are essential for students’ mental health. Insufficient university support has been reported. We call on educators and academic institutions to play more proactive roles in identifying those who require assistance. Academic institutions should adopt a holistic approach to education and provide student-oriented services to meet students’ learning, emotional and social needs and enhance their mental health.

## Figures and Tables

**Table 1:** Students’ characteristics (*n* = 1121).

**Demographic Characteristics**		***n* (%)**
Sex	Male	311 (27.7)
Age	18–24 years old	906 (80.8)
	25–29 years old	142 (12.7)
	30 years or older	73 (6.5)
Ethnicity	Chinese	1027 (91.6)
	Other Asian	67 (6.0)
	Non-Asian	27 (2.4)
Country or region of residence	Hong Kong	971 (86.6)
	Mainland China	113 (10.1)
	United States of America	1 (0.1)
	Canada	1 (0.1)
	Others	35 (3.1)
**Academic Programme Characteristics**		***n* (%)**
Mode of study	Full-time	975 (87.0)
	Part-time	146 (13.0)
Education programme level	Bachelor’s degree	860 (76.7)
	Master’s degree	197 (17.6)
	Doctoral Degree	53 (4.7)
Programme Year	First-year	345 (30.8)
	Non-first ad non-final year	510 (45.5)
	Final year	266 (23.7)
Field of study	Medical or health-related	485 (43.3)
	Others	636 (56.7)
Programme with A Practicum Component	Yes	555 (49.5)
	No	566 (50.5)
**Scores of Mental Health, Resilience, Family Functioning and Support System**		**Mean ± SD**
Anxiety symptom (GAD-2) ^1^		2.6 ± 1.5
Depression symptom (PHQ-2) ^2^		2.1 ± 1.5
Resilience (CD-RISC-2) ^3^		4.5 ± 1.5
Family functioning (BAFFS) ^4^		6.1 ± 1.8
Support from family		6.7 ± 2.6
Support from peers		6.3 ± 2.4
Support from universities		4.4 ± 2.5
**Level of Mental Health, Resilience, Family Functioning and Support System**		***n* (%)**
Anxiety symptoms (GAD-2) ^1^	Yes (3–6)	442 (39.4)
Depression symptoms (PHQ-2) ^2^	Yes (3–6)	365 (32.6)
Resilience (CD-RISC-2) ^3^	High (5–8)	539 (48.1)
Family functioning (BAFFS) ^4^	Distress (7–12)	388 (34.6)
Support from family ^5^	High (6–10)	792 (70.7)
Support from peers ^5^	High (6–10)	747 (66.6)
Support from Universities ^5^	High (6–10)	362 (32.3)

^1^ GAD-2: 2-item General Anxiety Disorder Screener, an anxiety subscale to screen for anxiety symptoms, range: 0–6; higher scores indicate more severe symptoms; range: 0–2, normal: 3–6, had anxiety symptoms; ^2^ PHQ-2: 2-item Patient Health Questionnaire—A depression subscale to screen for depression symptoms, range: 0–6; higher scores indicate more severe symptoms; range: 0–2, normal: 3–6, had depression symptoms; ^3^ CD-RISC-2: 2-item version of the Connor–Davidson Resilience Scale to assess resilience; higher scores indicate better adaptability, range: 0–8;0–4, low resilience, 5–8, high resilience; ^4^ BAFFS: 3-item Brief Assessment of Family Functioning Scale to evaluate family functioning; higher scores indicate greater distress, range: 3–12, 3–6, family satisfaction, 7–12, family distress; ^5^ Support from family, peers and universities; higher scores indicate better support, range: 0–10 for each item, 0–5, low support, 6–10, high support.

**Table 2:** Associations of students’ mental health with demographics and academic programme characteristics.

	Anxiety Symptoms				Depression Symptoms			
	Did Not Have Anxiety Symptoms	Had Anxiety Symptoms	OR, 95 % C.I. ^a^	*p*	They Did Not Have Depression Symptoms	Had Depression Symptoms	OR, 95 % taC.I. ^a^	*p*
	*n* (%)	*n* (%)			*n* (%)	*n* (%)		
Sex								
Male	194 (62.4)	117 (37.6)	0.90 (0.69, 1.18)	0.44	206 (66.2)	105 (33.8)	1.08 (0.82, 1.42)	0.59
Female	485 (59.9)	325 (40.1)			550 (67.9)	260 (32.1)		
Age								
18–24 years old	547 (60.4)	359 (39.6)	1.04 (0.77, 1.42)	0.78	603 (66.6)	303 (33.4)	1.24 (0.90, 1.72)	0.20
25 years old or above	132 (61.4)	83 (38.6)			153 (71.2)	62 (28.8)		
Ethnicity								
Chinese	636 (61.9)	391 (38.1)	0.52 (0.34, 0.79)	0.002 **	699 (68.1)	328 (31.9)	0.72 (0.47, 1.12)	0.14
Others	43 (45.7)	51 (54.3)			57 (60.6)	37 (39.4)		
Country or region of residence								
Hong Kong	593 (61.1)	378 (38.9)	0.86 (0.60, 1.21)	0.38	660 (68.0)	311 (32.0)	0.84 (0.58, 1.20)	0.33
Others	86 (57.3)	64 (42.7)			96 (64.0)	54 (36.0)		
Mode of study								
Full-time	588 (60.3)	387 (39.7)	1.09 (0.76, 1.56)	0.64	649 (66.6)	326 (33.4)	1.38 (0.93, 2.04)	0.11
Part-time	91 (62.3)	55 (37.7)			107 (73.3)	39 (26.7)		
Education programme level								
Undergraduate	512 (59.5)	348 (40.5)	1.21 (0.91, 1.61)	0.20	565 (65.7)	295 (34.3)	1.42 (1.05, 1.94)	0.02 *
Postgraduate	167 (64.0)	94 (36.0)			191 (73.2)	70 (26.8)		
Programme Year—First year								
Yes	200 (58.0)	145 (42.0)	1.17 (0.90, 1.51)	0.24	228 (66.1)	117 (33.9)	1.09 (0.83, 1.43)	0.52
No	479 (61.7)	297 (38.3)			528 (68.0)	248 (32.0)		
Programme Year—Final year								
Yes	169 (63.5)	97 (36.5)	0.85 (0.64, 1.13)	0.26	184 (69.2)	82 (30.8)	0.90 (0.67, 1.21)	0.49
No	510 (59.6)	345 (40.4)			572 (66.9)	283 (33.1)		
Field of study								
Medical or health-related	323 (66.6)	162 (33.4)	0.64 (0.50, 0.81)	<0.001 ***	335 (69.1)	150 (30.9)	0.88 (0.68, 1.13)	0.31
Others	356 (56.0)	280 (44.0)			421 (66.2)	215 (33.8)		
Programme with a practicum component								
Yes	345 (62.2)	210 (37.8)	0.88 (0.69, 1.11)	0.28	379 (68.3)	176 (31.7)	0.93 (0.72, 1.19)	0.55
No	334 (59.0)	232 (41.0)			377 (66.6)	189 (33.4)		

Analysis was conducted by logistic regression: * *p* < 0.05, ** *p* < 0.01, *** *p* < 0.001; ^a^ OR (95% CI) = odds ratio (95% confidence interval).

**Table 3:** Associations of mental health with the level of resilience, family functioning and support system.

	Anxiety Symptoms ^1^			Depression Symptoms ^2^		
	Normal	Had Anxiety Symptoms		Normal	Had Depression Symptoms	
	*n* = 679	*n* = 442		*n* = 756	*n* = 365	
	*n* (%)	*n* (%)	Adjusted OR (95% CI) ^a, b^	*n* (%)	*n* (%)	Adjusted OR (95% CI) ^a, b^
**Resilience (CD-RISC-2) ^3^**						
Low (0–4) (Reference)	292 (50.2)	290 (49.8)		324 (55.7)	258 (44.3)	
High (5–8)	387 (71.8)	152 (28.2)	0.39 (0.30, 0.50) ***	432 (80.1)	107 (19.9)	0.31 (0.23, 0.40) ***
**Family functioning (BAFFS) ^4^**						
Satisfaction (3–6) (Reference)	481 (65.6)	252 (34.4)		537 (73.3)	196 (26.7)	
Distress (7–12)	198 (51.0)	190 (49.0)	1.90 (1.47, 2.45) ***	219 (56.4)	169 (43.6)	2.15 (1.65, 2.79) ***
**Support from family ^5^**						
Low (0–5) (Reference)	181 (55.0)	148 (45.0)		201 (61.1)	128 (38.9)	
High (6–10)	498 (62.9)	294 (37.1)	0.67 (0.52, 0.88) **	555 (70.1)	237 (29.9)	0.66 (0.51, 0.87) **
**Support from peers ^5^**						
Low (0–5) (Reference)	195 (52.1)	179 (47.9)		227 (60.7)	147 (39.3)	
High (6–10)	484 (64.8)	263 (35.2)	0.58 (0.45, 0.75) ***	529 (70.8)	218 (29.2)	0.63 (0.49, 0.82) ***
**Support from universities ^5^**						
Low (0–5) (Reference)	445 (58.6)	314 (41.4)		487 (64.2)	272 (35.8)	
High (6–10)	234 (64.6)	128 (35.4)	0.77 (0.59, 1.0) *	269 (74.3)	93 (25.7)	0.62 (0.47, 0.83) ***

^1^ Measured by GAD-2: 2-item General Anxiety Disorder Screener, an anxiety subscale to screen for anxiety symptoms, range: 0–2, normal: 3–6, had anxiety symptoms; ^2^ Measured by PHQ-2: 2-item Patient Health Questionnaire, a depression subscale to screen for depression symptoms, range: 0–2, normal: 3–6, had depression symptoms; ^3^ Measured by CD-RISC-2: 2-item version of the Connor–Davidson Resilience Scale to assess resilience; higher scores indicate better adaptability, range: 0–8, 0–4, low resilience, 5–8, high resilience; ^4^ Measured by BAFFS: 3-item Brief Assessment of Family Functioning Scale to evaluate family functioning; higher scores indicate greater distress, range: 3–12, 3–6, family satisfaction, 7–12, family distress; ^5^ Support from family, peers and universities; higher scores indicate better support, range: 0–10 for each item, 0–5, low support, 6–10, high support; ^a^ between-group differences of variables with adjustment of sex, age, ethnicity, education programme level, and field of study; ^b^ OR (95% CI) = odds ratio (95% confidence interval); * *p* = 0.05, ** *p* < 0.01, *** *p*< 0.001.

**Table 4:** Associations of resilience with family functioning and support system.

	Resilience ^1^			
	R ^a^	*p*	Adjusted r ^b^	*p*
Family functioning ^2^	−0.15	<0.001 ***	−0.15	<0.001 ***
Support from family ^3^	0.20	<0.001 ***	0.21	<0.001 ***
Support from peers ^3^	0.16	<0.001 ***	0.17	<0.001 ***
Support from universities ^3^	0.19	<0.001 ***	0.19	<0.001 ***

^1^ measured by CD-RISC-2: 2-item version of the Connor–Davidson Resilience Scale to assess resilience; higher scores indicate better adaptability, range: 0–8; ^2^ measured by BAFFS: 3-item Brief Assessment of Family Functioning Scale to evaluate family functioning; higher scores indicate greater distress, range: 3–12; ^3^ Support from family, peers and universities; higher scores indicate better support, range: 0–10 for each item; ^a^ Pearson correlation without adjustments for confounding variables; ^b^ Partial correlation adjusting for sex, age, ethnicity, education programme level, and field of study; *** Correlation is significant (2-tailed), *p* < 0.001.

## Data Availability

The data supporting this study’s findings are available from the corresponding author (A.Y.K.L.) upon reasonable request.
